# Editorial Comment from Dr Kato to Rare case of a patient with testicular torsion complicated by acute pneumonia, requiring emergency surgery, during the COVID‐19 pandemic

**DOI:** 10.1002/iju5.12410

**Published:** 2021-12-26

**Authors:** Manabu Kato

**Affiliations:** ^1^ Department of Nephro‐Urologic Surgery and Andrology Mie University Graduate School of Medicine Tsu Mie Japan

Coronavirus disease‐2019 (COVID‐19) has been prevalent in Japan since April 2020. Since then, we have experienced five major infectious waves so far. Meanwhile, a state of emergency was announced in a major city and almost all prefectures in Japan, greatly impacting daily life. Or rather, the traditional daily lifestyle has transitioned to a newer lifestyle. This new lifestyle contains diverse action recommendations, including to avoid close contact, to wear masks both outside and inside environments, restrictions relating to working hours of restaurants at night especially offering alcoholic beverages, business work styles (the increase of remote work), the guidance and encouragement of vaccinations, and even a change in the consultation styles at medical institutions. This change in consultation style includes mandatory COVID‐19 antigen testing for emergency patients (including regular inpatients scheduled for general anesthesia). Since the COVID‐19 antigen test takes at least 20–30 min after the first visit, treatment for severe disease conditions such as operations for testicular torsion, urgent renal fistula construction for acute renal failure, urgent bladder fistula construction for urinary retention, and ureteral catheter placement for urinary stone pyelonephritis may be delayed. The dilemma that becomes evident is that treatment prognoses may be affected. It is clear in emergency situation, but it also could happen in cancer treatment. In fact, multiple reports indicate that delaying treatment for conditions by checking incoming patients for COVID‐19 leads to a poorer outcome of cancer treatment.^1^, ^2^

This report describes a situation in which a urological emergency necessitating surgical intervention for testicular torsion was slightly delayed due to the coexistence of testicular torsion and pneumonia suspected from COVID‐19.^3^ Fortunately, the testes were preserved. If the diagnosis of pneumonia was delayed, however, the testes may not have been preserved. Some reported urology standard of care during the COVID‐19 pandemic.^4^ What we need to learn from this case report is that situations requiring immediate medical intervention could have poorer outcomes if treatments were delayed for testing for COVID‐19 infection.

In this COVID‐19 era, it becomes very important for emergency general hospitals treating urological conditions to be prepared for the treatment of patients with suspected COVID‐19 or suspected coexistence of this type of pneumonia.

## Conflict of interest

The author declares no conflict of interest.

## References

[1] Hamilton W . Cancer diagnostic delay in the COVID‐19 era: what happens next? Lancet Oncol. 2020; 21: 1000–2. 32702312 10.1016/S1470-2045(20)30391-0 PMC7834491

[2] Maringe C , Spicer J , Morris M *et al*. The impact of the COVID‐19 pandemic on cancer deaths due to delays in diagnosis in England, UK: a national, population‐based, modelling study. Lancet Oncol. 2020; 21: 1023–34. 32702310 10.1016/S1470-2045(20)30388-0 PMC7417808

[3] Arai M , Okada Y , Takeshita H *et al*. A rare case of a patient with testicular torsion complicated by acute pneumonia, requiring emergency surgery, during the COVID‐19 pandemic. IJU Case Rep. 2022; 5: 99–101. 10.1002/iju5.12404 PMC8888013 35252789

[4] Heldwein FL , Loeb S , Wroclawski ML *et al*. A systematic review on guidelines and recommendations for urology standard of care during the COVID‐19 pandemic. Eur. Urol. Focus 2020; 6: 1070–85. 32532703 10.1016/j.euf.2020.05.020 PMC7274599

